# Selective killing of breast cancer cells expressing activated CD44 using CD44 ligand-coated nanoparticles *in vitro* and *in vivo*

**DOI:** 10.18632/oncotarget.3681

**Published:** 2015-03-29

**Authors:** Cuixia Yang, Yiqing He, Huizhen Zhang, Yiwen Liu, Wenjuan Wang, Yan Du, Feng Gao

**Affiliations:** ^1^ Department of Molecular Biology Laboratory, Shanghai Sixth People's Hospital, Shanghai Jiaotong University, Shanghai, China; ^2^ Department of Clinical Laboratory, Shanghai Sixth People's Hospital, Shanghai Jiaotong University, Shanghai, China; ^3^ Department of Pathology, Shanghai Sixth People's Hospital, Shanghai Jiaotong University, Shanghai, China

**Keywords:** CD44, breast cancer, selective targeting therapy, hyaluronan

## Abstract

The cell surface glycoprotein CD44 is expressed in cancer cells and has been used as a therapeutic target in preclinical studies. However, the ubiquitous expression of CD44 in numerous cell types, including hematopoietic cells, has hindered its application in targeted therapy. Here, we demonstrated that CD44 was activated on breast cancer cells but was inactive on normal cells *in vitro* and *in vivo*. We analyzed 34 clinical primary tumor and normal breast tissues and demonstrated that CD44 was in an active state on breast cancer cells but in an inactive state on normal cells. Furthermore, based on the binding property of CD44 with its ligand hyaluronan (HA), we self-assembled HA-coated nanoparticles and studied their selective targeting efficacy. Our results indicate that HA-coated nanoparticles bearing the CD44 ligand selectively targeted cancer cells both *in vitro* and *in vivo*, killing breast cancer cells while sparing normal cells. Our study suggested that the active state of CD44 plays a crucial role in the selective targeting of breast cancer cells by avoiding nonspecific toxicity to CD44-quiescent normal cells. These findings may provide a new idea for the selective targeting of cancer cells in other human cancers.

## INTRODUCTION

Breast cancer remains a leading cause of morbidity and mortality in women worldwide, and the selection of proper treatment for an individual with a specific type of breast cancer is urgently needed to improve survival [1, 2]. Although new specific targets for breast cancer treatment are rapidly emerging, many problems remain to be resolved. It is likely that the most common and serious obstacle regarding current treatments is low target specificity, which drastically influences the efficacy and adverse effects. CD44, a cell-surface glycoprotein, is highly expressed in tumors, especially in breast cancer [3, 4]. Along with the development of humanized CD44 antibodies by multiple pharmaceutical companies, recent preclinical and clinical trials of anti-CD44 therapy have become possible (Patent Nr. 7507537, Patent Nr. 5916561). At present, the most effective strategy for CD44 targeted anti-cancer therapeutics is anti-CD44 antibodies. The most exciting news to come from such strategies is that a CD44 antibody-drug conjugate exhibited clinical successes in both metastatic breast cancer and squamous cell carcinoma (SCC) [5, 6]. Despite these promising results, CD44 targeting was not completely successful in clinical practice [7, 8], and clinical trials involving anti-CD44 antibody were halted due to various toxicities [5, 7].

Consistent with clinical limitations, the laboratory results for CD44 targeting are not convincing. As mentioned above, in addition to being over-expressed in tumors, CD44 is also widely expressed in almost every cell type in humans and mice [9, 10]. Initial trials with drugs conjugated to CD44 antibodies are complicated by various toxicities, which are partly due to the fact that anti-cancer drugs conjugated to a CD44 antibody may induce toxicity in both cancer and normal cells. Therefore, the effectiveness and safety of CD44 in targeting chemotherapy must be clarified.

The main ligand for the CD44 receptor is hyaluronan (HA), a glycosaminoglycan that is synthesized in a wide range of sizes up to ~10^7^ Da. HA could act as a targeting carrier coating on nanoparticles because it is a water soluble, non-immunogenic polysaccharide with multiple functional groups available for chemical conjugation. HA reduces systemic toxicity by delivering a higher relative dose to tumors in mice [11, 12]. However, most of these studies focused on the chemical properties of HA and underestimated its potential to selectively target CD44.

HA acts through CD44 to regulate cell proliferation and motility. The interaction of CD44 with HA is strongly influenced by the state of CD44 activation [13, 14]. As previously reported, CD44 exists in two distinct states that differ in HA-binding affinity: active or inactive [15]. However, many examples indicate that CD44-positive cells do not bind HA, whereas only activated CD44 binds and internalizes HA. Inactive CD44 can not bind HA unless it is activated by appropriate stimuli, such as numerous cytokines, growth factors, and alterations in the cellular context [16, 17]. Many types of cells express CD44 at various levels, yet HA binding is not detectable [18] or exclusively stimulated under particular conditions [19]. These reports suggest that CD44 activation at appropriate times and locations might play a vital role in biological processes, such as cancer progression, and could therefore be used as a specific target in anti-cancer therapy. However, the role of activated CD44 in previous targeting therapy efforts was not sufficiently addressed. Thus, it is important to identify differences in CD44 ligand binding capacities of cancerous and normal tissues or cells.

In this study, we aimed to investigate the role of CD44 activation states in targeting therapy to breast cancer cells. We determined that two different states of CD44 exist between human breast cancerous and normal cells from pathological specimens and cell lines. Self-assembling HA-coated nanoparticles were prepared to selectively target tumor cells with activated CD44 *in vivo*. Our study suggests that CD44 on cancer tissues and cancer cells is considerably more active compared with CD44 from normal control cells and that this activation plays a crucial role in selectively targeting therapy to breast cancer cells.

## RESULTS

### Different activation states of CD44 in clinical normal and malignant breast tissues

**Figure 1:**
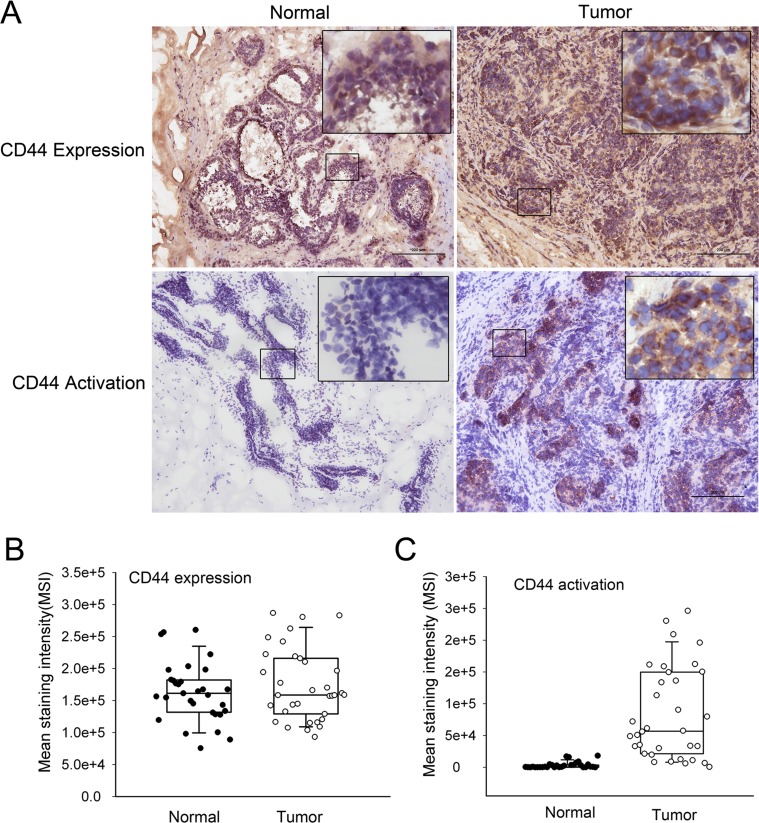
CD44 activation states in normal and cancerous breast tissues (**A**) CD44 expression and activation were determined by immunohistochemical analysis. Representative immunohistochemical findings of normal breast tissue and invasive ductal carcinoma of the breast using anti-CD44H mAb and fl-HA. Both normal and cancerous tissues express high levels of CD44 (left). CD44 positivity in normal breast tissue is restricted to the cell membrane of myoepithelial cells, whereas invasive carcinoma nests primarily exhibit positivity at cell membranes. The tumor specimens exhibited increased fl-HA binding, with fl-HA binding noted in both the tumor epithelium and lymphoid infiltrate. However, normal tissues exhibited minimal fl-HA binding. (**B**) The distribution of CD44 expression in 34 paired tumor and normal adjacent tissues samples (*P* > 0.05). (**C**) The distribution of the expression of activated CD44 in 34 paired tumor and normal adjacent tissues samples (*P* < 0.05). Whiskers indicate the 10th and 90th percentiles; ‘boxes’ mark 25th and 75th percentiles.

CD44 expression and activation states were analyzed in 34 frozen normal and cancerous breast tissue sections. Breast carcinoma tissues were surgically resected, and normal breast tissues were obtained from patients who had undergone reduction mammoplasties. The pathologic characteristics of this series of carcinomas are presented in Table 1. Immunohistochemical analysis demonstrated that CD44 was expressed on both cancerous and normal tissue samples (Fig. 1A). The MSI for CD44 detected on the surface of normal breast tissues (median: 160031.17) did not significantly differ from that of the MSI for cancerous tissues (median: 158634.25) (Fig. 1B).

**Table 1:** Clinical and pathological features of study population in relation to CD44-HA binding activity

Clinical parameters	CD44-HA binding activity			
	Number	Median	Range	*P* value
**Histologic Grade**				
1	5	149764	695-230440	0.932
2	17	57438	6011-246371	
3	12	49910.5	12660-161665	
**Histologic I type**				
Invasiveduct carcinoma	30	57438	695-246371	0.553
Non-invasive Duct carcinoma	4	41173	6011-16665	
**Tumor size**				
T1	6	73381	21332-162404	0.466
T2	17	43162	695-230440	
T3	11	158863	8187-246371	
**Lymph node metastasis**				
No	25	56231	695-230440	0.701
Yes	9	57438	8187-246371	
**Age**				
<50year	10	34587.5	6011-136276	0.170
≥50year	24	76013.5	695-979883	
**ER receptor**				
(−)	13	61512	8843-196248	0.462
(+)	21	56231	695-246371	
**PR receptor**				
(−)	19	61512	8187-209222	0.451
(+)	15	56231	695-246371	
**Her 2 receptor**				
(−)	7	79885	695-196248	0.934
(+)	27	56231	6011-246371	
**Ki 67**				
<20%	12	58871.5	695-230440	0.986
≥20%	22	54185.5	2830-246371	

ER, estrogen receptor; PR, progesterone receptor

To evaluate CD44 activity in clinical samples, we used the affinity probe biotin-labeled HA, which only binds to CD44 in its active conformation [24]. The results demonstrated increased HA binding on cancerous tissues with a median MSI of 56834.5 compared with minimal or low binding on the corresponding normal tissue with a median MSI of 1.9795 (Fig. 1A and 1C), indicating the presence of two distinct states of CD44 activity determined by microenvironments.

Furthermore, we examined the relationship between activated CD44 and tumor grade, histological type, tumor size, lymph node metastasis stage, age at diagnosis, estrogen receptor status, progesterone receptor, Her 2 receptor, and Ki 67. Despite the obvious differences between cancerous and normal breast tissues, no significant association was observed between activated CD44 expression and these tumor characteristics (Table 1), indicating no correlation between CD44 activation and clinical tumor parameters. In addition, further examination revealed that alterations of CD44 states can occur within the breast tumor microenvironment (Fig. S1). HA binding of CD44 on normal cells was observed when normal cells were co-cultured with breast cancer cells, indicating that the conversion of CD44 from an inactive state to an active state (Fig. S1). This result provides evidence for the specific location of active CD44 within the tumor microenvironment.

### Two CD44 activation states in normal and breast cancer cell lines

**Figure 2:**
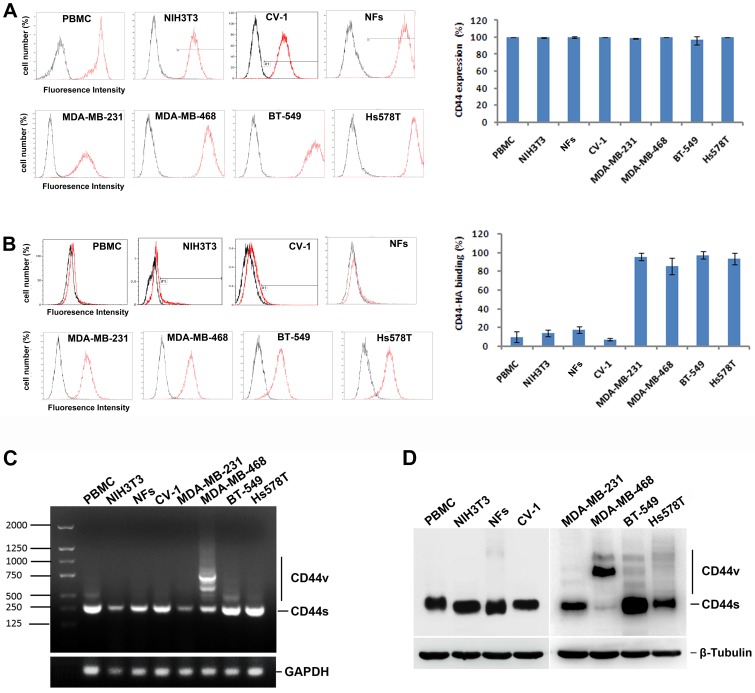
Two activation states of CD44 in normal and breast cancer cell lines (**A**) CD44 expression on four breast cancer cell lines (MDA-MB-231, MDA-MB-468, BT-549, and Hs578T) and four normal cells (PBMC, NIH3T3, CV-1, and NFs) were determined by flow cytometry. (**B**) The binding activity of HA by CD44 on four breast cancer cell lines (MDA-MB-231, MDA-MB-468, BT-549, and Hs578T) and four normal cells (PBMC, NIH3T3, CV-1, and NFs) were determined and analyzed. (C) The CD44 isoform expression pattern was detected by agarose gel electrophoretograms in normal cells and cancer cells. (**D**) The CD44 variants expression was detected by western blot in normal cells and cancer cells.

To further confirm that the two activation states of CD44 are differentially located, we assessed CD44 expression and fl-HA binding activity on four breast cancer cell lines (MDA-MB-231, MDA-MB-468, BT-549, and Hs578T) and four normal cell lines (PBMCs, NIH3T3, CV-1, and NFs). The peripheral blood mononuclear cells (PBMCs) from 10 donors and NFs derived from 3 adults were assessed. The results from flow cytometry analysis indicated that CD44 expression was abundant in all four breast cancer cell lines and four normal cell lines; no significant difference between normal and cancer cells was noted (Fig. 2A). However, significantly increased fl-HA binding was observed in cancer cell lines compared with normal cells (Fig. 2B). These results indicated that the CD44 activation state differs in normal and cancer cells, revealing striking similarities to the observations made in clinical tissues.

To determine whether the CD44 expression pattern is altered in these four breast cancer cell lines compared with the four normal cells, we next determined the expression of CD44 variants. Using a primer pair spanning the entire variant region in a reverse transcription polymerase chain reaction (RT-PCR) assay, each transcribed CD44 variant isoform is theoretically amplified as described previously [25]. Our results indicate that the CD44 expression pattern differed between normal cells and cancer cells (Fig. 2C). Human breast cancer cell lines (MDA-MB-468, BT-549, and Hs578T) were characterized by the appearance of several bands on agarose gel electrophoretograms when examining the CD44 variant region with a primer pair spanning the entire variant region. These results were consistent with previous reports [26]. By contrast, almost no expression of the CD44v gene was observed in normal cells (Fig. 2C). Similarly, changes in expression patterns were detected in normal cells and cancer cells by western blotting using an immunoblotting antibody against the standard region of CD44 (Fig. 2D). Although altered CD44 mRNA and protein expression patterns were detected in three of four cancer cell lines tested, which might be attributed to the heterogeneity of cancer cell lines, no obvious difference was noted regarding the fl-HA binding activity of the four cancer cell lines as analyzed in Fig. 2B, suggesting that factors other than CD44v expression affect the different CD44 activation states noted between normal and cancer cells.

### Engineering and synthesis of HA-coated nanoparticles

To develop HA-coated nanoparticles containing drugs, a nanotechnology engineering approach was employed based on structural and functional studies of HA as described previously [21, 27]. Paclitaxel (PTX) was mixed with the lipid molecules and assembled into lipid clusters, which were subsequently covalently coated with HA to form HA-Lipid-PTX nanoparticles. HA-free Lipid-PTX nanoparticles were prepared similarly with the omission of HA. The particle size distribution and polydispersity index (PDI) were determined by light scattering using particle-sizing systems. HA-free nanoparticles (Lipid-PTX) exhibit size ranges of 100 nm in diameter with a PDI of 0.118, and HA-coated nanoparticles exhibit size ranges of 120.5 nm in diameter with a PDI of 0.160. These values are within the range of those previously reported for paclitaxel carriers [28].

**Figure 3:**
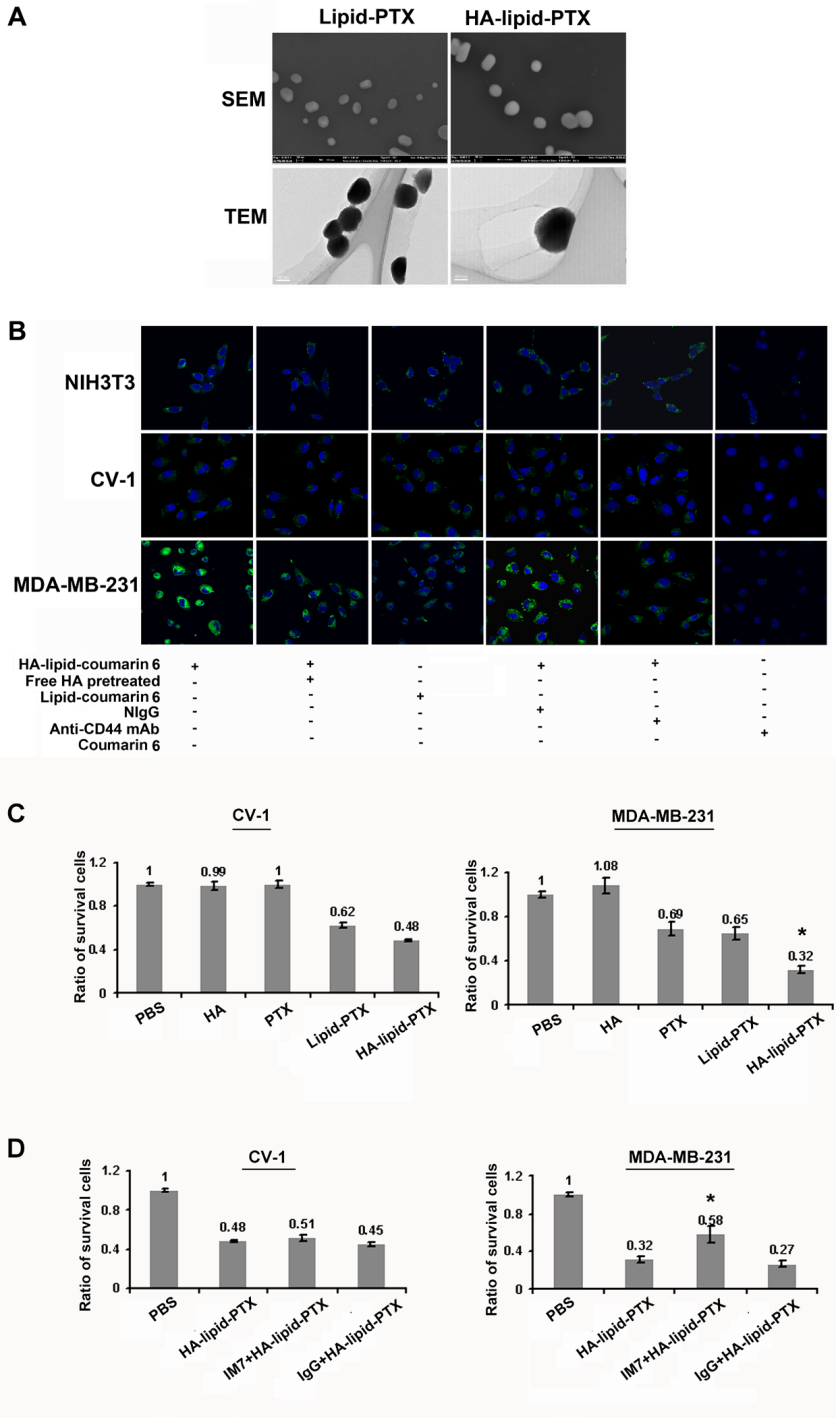
Activated CD44-mediated selective targeting of HA-coated nanoparticles (**A**) Characteristics of hyaluronan (HA)-coated nanoparticles. Morphology of HA-coated nanoparticles was measured using scanning electron microscopy (SEM) and transmission electron microscopy (TEM). Representative SEM and TEM images of the ultrastructure of lipid-PTX particles and HA-Lipid-PTX particles are presented. The globular shapes of the particles were obvious. (**B**) Confocal microscopic images of normal fibroblast (NIH3T3) and breast cancer cells (MDA-MB-231) incubated for 1 h with lipid-coumarin 6 and HA-lipid-coumarin 6. Images depict cytoplasmic localization of coumarin 6 (green). Blue indicates DAPI nuclear staining. Pre-treatment with free HA to block activated CD44 on the cell surface, and cellular internalization of HA-nanoparticles was highly decreased in MDA-MB-231 cells, almost to the extent of the blocking CD44 antibody. The normal IgG (NIgG), coumarin 6 and HA free Lipid-coumarin 6 were used as negative controls. (C, D) Selective cytotoxicity of cancer cells using HA-coated nanoparticles. (**C**) MTT assay to determine the viability of CV-1 cells and MDA-MB-231 cells treated with free HA, PTX, Lipid-PTX or HA-Lipid-PTX for 24 h. All results are expressed as the means ± SD (standard deviation) from three independent experiments performed in quintuplicate. *P < 0.05 compared with Lipid-PTX. (**D**) The effects of CD44 blockade via an anti-CD44 mAb on cytotoxicity. Pre-treatment with the CD44 blocking antibody (IM7) had no obvious effects on the cytotoxicity of HA-Lipid-PTX nanoparticles in CV-1 cells. Pre-treatment with the CD44 blocking antibody inhibited the cytotoxicity of HA-coated PTX in MDA-MB-231 cells. Normal IgG (NIgG) was used as a negative control. All results are expressed as the mean ± SD from three independent experiments performed in quintuplicate. *P < 0.05 compared with Lipid-PTX.

Lipid-PTX clusters and HA-Lipid-PTX exhibited globular shapes as evidenced by scanning electron microscopy (SEM) and transmission electron microscopy (TEM) images (Fig. 3A). In addition, the Zeta potentials of Lipid-PTX clusters and HA-Lipid-PTX nanoparticles were −12 mV and −29 mV, respectively, suggesting that the clustering structure is stabilized by HA coating.

### Activated CD44 directly induces the internalization of HA-coated nanoparticles

The induction of internalization was analyzed using a confocal imaging system after incubating the cells with HA-coated nanoparticles using a fluorescent marker (coumarin 6) as a model drug. CD44-active breast tumor cells (MDA-MB-231) exhibited more efficient internalization of the HA-coated nanoparticles compared with CD44-inactive normal cells (NIH3T3, CV-1) (Fig. 3B). These results indicate that HA-coated nanoparticles selectively bind to activated CD44 compared with inactive CD44. Moreover, the compartmentalization of fluorescent particles in intracellular vesicles of tumor cells provides clear evidence for successful endocytosis of HA-coated nanoparticles.

Additionally, after pre-treatment with free HA to block the binding site of activated CD44 on the cell surface, cellular internalization of HA-coated nanoparticles was significantly decreased, and the level of inhibition were similar to those observed with CD44 antibody pre-treatment in MDA-MB-231 cells (Fig. 3B). These results suggest the specific binding of HA-Lipid-PTX to active CD44 on the cancer cell surface. However, pre-treatment with free HA or the blocking CD44 antibody exhibited no obvious effects on the internalization of nanoparticles in CD44-inactive NIH3T3 and CV-1 cells. The differential uptake efficiency of HA-coated nanoparticles observed between normal and cancer cells is potentially induced by activated CD44.

### Activated CD44 selectively increases cytotoxicity in breast cancer cells

To examine whether HA-coated nanoparticle treatment selectively increases cytotoxicity in breast cancer cells via activated CD44, an MTT (3-(4, 5-dimethylthiazolyl-2)-2, 5-diphenyltetrazolium bromide) assay was performed to assess *in vitro* cytotoxicity. Normal cells (CV-1) and breast cancer cells (MDA-MB-231) were treated with HA-Lipid-PTX and Lipid-PTX. The results indicate that breast tumor cells (MDA-MB-231) with active CD44 appeared more sensitive to the cytotoxic activity of HA-coated nanoparticles than CD44-inactive normal cells (CV-1) (Fig. 3C). MDA-MB-231 cell growth was hindered, indicative of severe cytotoxicity. By contrast, CV-1 cell growth was largely unaffected, indicating only minor cytotoxicity. HA-free nanoparticles demonstrated low cytotoxicity in each cell line. Cells treated with an equal dose of HA or free PTX exhibited minimal effects on growth in each cell line (Fig. 3C).

To further define the role of activated CD44 in cell cytotoxicity, a blocking experiment on CD44 binding with HA was performed. Treatment with an anti-CD44 mAb strongly decreased HA-coated nanoparticle-induced cell cytotoxicity in MDA-MB-231 cells, but no obvious effects were noted in CV-1 cells (Fig. 3D), suggesting that activated CD44 mediates the increase in breast cancer cell selective cytotoxicity.

### Activated CD44 mediates selective targeting *in vivo*

**Figure 4:**
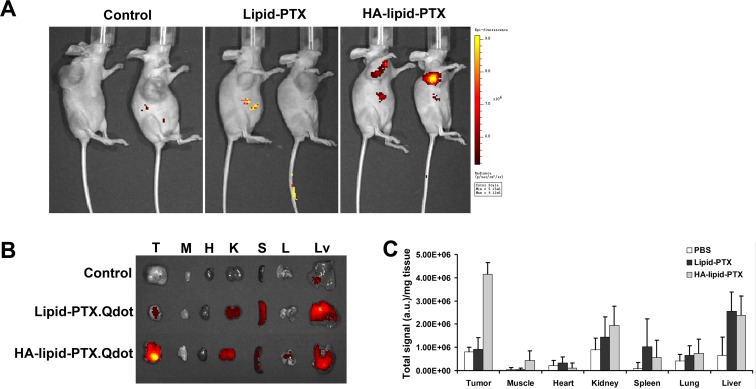
Selective targeting of activated CD44 *in vivo* (**A**) Optical imaging of mice administered either HA-coated nanoparticles carrying Qdot800 (targeted activated CD44) or HA-free nanoparticles (non-targeted nanoparticles). A high-intensity Qdot800 signal was exclusively detected in the tumor region exposed to the HA-coated nanoparticles (black arrow). The scale is in photons s-1 sm-2 sr-1. (**B**) Fluorescence images of normal organs and tumors collected at 24 h post-injection of HA-coated nanoparticles. Organ images are denoted as follows: T, tumor; M, muscle; H, heart; K, kidney; S, spleen; L, lung; Lv, liver. (**C**) Relative organ uptakes of Qdot800-labeled HA-coated nanoparticles in tumor-bearing mice 24 h post tail-vein injection. A relatively high level of Qdot800 accumulation occurs in tumor tissue. Error bars indicate SD; n = 3

*In vivo* imaging was used to illustrate the selective targeting of HA-coated nanoparticles to activated CD44 but not resting CD44 in normal tissues *in vivo*. Qdot800-labeled HA-coated nanoparticles were injected into veins of mice bearing MDA-MB-231 xenografts. This injection caused an intense fluorescent appearance of the Qdot800 at the tumor site (Fig. 4A), mostly likely due to specific binding of HA to activated CD44 in the xenografts. Under identical conditions, systemically administered HA-free control nanoparticles (Lipid-PTX.Qdot800) did not accumulate at the tumor site (Fig. 4A). PBS-treated mice bearing MDA-MB-231 xenografts were used as negative controls.

Organ-specific biodistribution was analyzed to further assess the effect of activated CD44 on the targeting selectivity of HA-coated nanoparticles *in vivo*. For these experiments, 24 h after the administration of nanoparticles coated with or without HA, considerably stronger signals were detected in the tumor regions of mice administered a HA-coated nanoparticles injection compared with mice receiving a HA-free nanoparticle injection (Fig. 4B and 4C). Notably, the relative organ uptake of HA-coated nanoparticles in tumors 24 h post-injection is strikingly increased compared with other organs (Fig. 4C), thus demonstrating effective targeting mediated by activated CD44 *in vivo*.

### Activated CD44 sensitizes breast cancer to drug treatment *in vivo*

**Figure 5:**
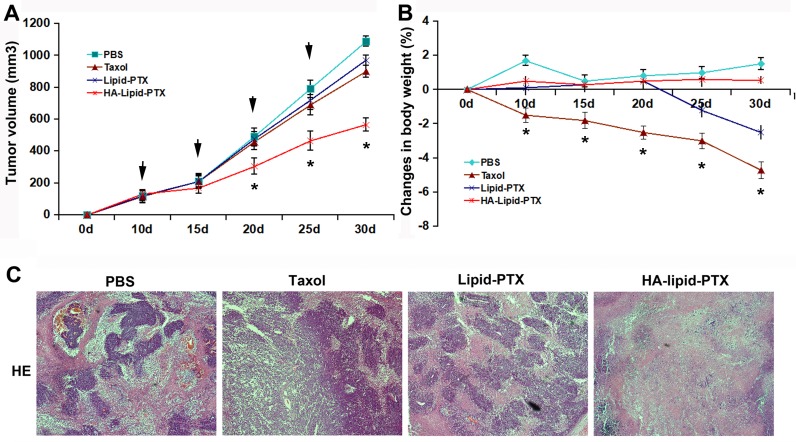
Anti-tumor effect of selectively targeted HA-coated nanoparticles *in vivo* The agents were i.v injected into xenografted mice on days 10, 15, 20, and 25 (n =5/group). The tumor volume (**A**) and body weight (**B**) of these mice were monitored. *P < 0.05 vs. taxol treatment. (**C**) Histological analysis of tumor tissues. H&E staining indicated increased acellular material in the HA-Lipid-PTX treatment group.

We next investigated whether the treatment of breast tumors with HA-coated nanoparticles inhibits xenograft growth and sensitize xenografts to paclitaxel treatment. HA-Lipid-PTX, Lipid-PTX, taxol, or PBS was administered intravenously into mice with MDA-MB-231 cell xenografts 4 times every 5 days. The group with HA-lipid-PTX treatment exhibited significant reduced tumor volume of xenografts compared with mice treated with PBS, HA, taxol, or Lipid-PTX at 20, 25 and 30 days (Fig. 5A).

A decrease in body weight is considered a global marker of toxicity. Therefore, we evaluated weight changes in mice after intravenous administration of the nanoparticles. HA-Lipid-PTX nanoparticles were well tolerated and did not cause any significant decrease in body weight or obvious skin toxicity (Fig. 5B). In comparison, mice treated with taxol exhibited reduced body weight (up to 5% by day 25). All mice were sacrificed on day 30, and tumors were excised for volume measurement and histological analysis. The tumors from mice in the HA-Lipid-PTX treatment group were obviously smaller compared with other groups (Fig. 5A). Histological analyses by hematoxylin and eosin (H&E) staining provided additional confirmation that the HA-coated nanoparticles exhibited marked selective cytotoxicity to tumor cell xenografts. H&E staining revealed that tumor sections from the HA-Lipid-PTX treatment group were considerably less cellular, whereas tumor sections from other groups contained tightly packed cells (typical of adenocarcinomas) interspersed with blood sinuses (Fig. 5C).

## DISCUSSION

Anti-CD44 antibodies have been exploited for targeted delivery of anti-cancer agents to several types of carcinomas, including breast, colorectal, and lung cancers [29, 30]. Although these antibodies exhibited some promise, drugs conjugated to CD44 antibodies caused various toxicities in human patients in initial trials [7, 8], largely limiting their utility in the clinic. The main toxicity of mAb-conjugates was directed against skin and hematologic cells [31, 32], most likely due to CD44 expression in these cells. Therefore, the wide expression of CD44 in normal tissues is a complicating factor in CD44 antibody-conjugated drug-induced toxic epidermal necrolysis and hematologic toxicity, thus making selective and safe antibody-based cancer therapy impossible. Thus, selectively targeting CD44 in tumor cells is crucially needed for this approach to work.

Our experiments regarding the different states of CD44 activation demonstrate that CD44 is active on cancerous breast cells but inactive on normal cells. Our unique data indicate that selective targeting mediated by HA is dependent on the distinct activation state of CD44. It is plausible that only active CD44 possesses the capability to bind with its ligand HA and that inactive CD44 cannot bind HA [13, 24]. Previously published studies on CD44 expression and CD44-mediated internalization are riddled with paradoxes and apparent contradictions. Some reports suggested that the amount of HA internalized in many cell lines is not directly proportional to CD44 receptor density, and cells that exhibited 10-fold increased CD44 receptor expression internalized similar amounts of HA-conjugate [27]. However, until now, the function of CD44 and its activation state in the properties of these particular cells have received little attention.

In this study, we demonstrate that two distinct CD44 states exist and that these states differ in human breast cancerous and normal tissue: active CD44 is found on cancer cells and inactive CD44 is found on normal cells. All patients with primary invasive ductal carcinoma of the breast (stage I-III) were diagnosed pathologically and clinically, and the specimens were obtained before any treatments were administered. The results indicate high CD44 expression on both breast cancer tissues and normal control tissues. However, the HA-binding abilities of CD44 were significantly higher for cancer tissues compared with normal tissues, strongly suggesting that a discordant relationship of CD44 expression and activation exists between cancerous and normal breast tissues. Our results also demonstrate that quiescent CD44 on normal cells could be activated in tumor microenvironments (Fig. S1 in S1 Supplemental Data), providing additional evidence for the specific active state of CD44 on tumor cells. However, the mechanisms for this are not clear. Previous studies demonstrated that cancer cells typically produce several variant forms of CD44 in addition to the standard CD44, whereas some tumor types, e.g., gliomas, produce mainly the standard form [33]. Other works indicated that variants of CD44 are significantly amplified in numerous tumor types compared with normal processes [34]. However, all forms of CD44 include an N-terminal, membrane-distal, hyaluronan-binding domain that exhibits significant homology with other forms, harboring similar features of “activation” for HA-CD44 interaction. Our HA-CD44 binding experiments covered all variants of CD44 for native HA can bind to any activated CD44 variant.

Following findings in human breast tissues, we performed similar experiments using cultured cells to further identify the differences regarding CD44 active states. Among the eight cell lines tested, most of the cells expressed high levels of CD44. However, the HA-binding capacities significantly differed. Breast cancer cells exhibited significantly increased binding activity compared with normal cells, and these results are consistent with the results from patient-derived tissues. Moreover, our data revealed that the expression patterns of variant CD44 differed between normal and cancer cells and that cancer cells were more likely to express CD44v isoforms than normal cells. CD44v isoforms are implicated in drug resistance [35], and their expression correlates with adverse outcomes in patients with breast [36], colon [29] and gastric carcinoma [37]. However, in the four cancer cells we assessed, CD44v expression patterns exhibited a high degree of heterogeneity and were not associated with HA binding capacities, suggesting that CD44v expression might not serve as the main cause of the different CD44-activation states noted in normal and cancer cells. Fluorescence microscopy analysis revealed a high level of internalization of the HA-coated nanoparticles by breast cancer cells, whereas minimal internalization was noted in normal fibroblast cells. These results suggest that HA-coated nanoparticles can distinguish between the different states of CD44 on normal and cancerous cells. Furthermore, HA-coated nanoparticles induced enhanced cytotoxicity in breast cancer cells, whereas no obvious effects were noted in normal cells. These results strongly suggest that the unparalleled phenomenon of CD44 expression and activation might promote selective CD44 targeting therapy. It is likely that HA distinguishes activated CD44 on tumor cells from normal cells, and the use of HA as a selective targeting carrier might serve as a promising avenue for systemic anti-cancer therapeutics.

As the main ligand for CD44, HA was used as a drug carrier coating on liposomes or nanoparticles to deliver drugs [27]. However, for the HA-associated anti-cancer study, most of the studies focused on the chemical properties of HA [12, 38]. Few studies have attempted to investigate the features of CD44. In this study, the targeting of HA-coated nanoparticles was compared between cancerous and normal cells *in vitro* and *in vivo*. The data suggested that this carrier system potentially represents a promising candidate for selectively targeted drug delivery to the tumor rather than adjacent normal tissues. To further confirm the differential targeting efficiency of HA-coated nanoparticles between normal and cancerous cells, we performed an *in vivo* experiment wherein a breast cancer xenograft model was established in nude mice, and HA-Lipid-PTX nanoparticles were administered systemically. The HA-Lipid-PTX biodistribution profile *in vivo* exhibited significantly increased uptake by the tumor compared with HA-free nanoparticles. In addition, mice with high CD44 expression and active breast cancer cells displayed smaller tumor volumes and increased cellular necrosis after HA-Lipid-PTX treatment, suggesting it is a considerably more effective treatment strategy. By contrast, no obvious cytotoxicity was observed in other organs. The results suggested that HA-Lipid-PTX induces severe cytotoxicity in CD44-active human breast cancer cells *in vivo* and exerts no effect on normal cells and organs. Therefore, this study suggests that the HA-coated carrier system might represent a promising candidate for selectively targeted drug delivery to CD44-active tumors rather than CD44-quiescent adjacent normal tissues.

In summary, this study demonstrates a novel approach for selective therapy by nanoparticle targeting mediated through the interaction of HA with activated CD44. The selective toxicity of HA-coated nanoparticles for breast cancer cells demonstrated in this study should encourage the clinical evaluation of this nanoparticle in the treatment of breast cancer patients.

## MATERIALS AND METHODS

### Reagents

FITC-CD44 mAb (clone IM7) for flow cytometry analysis was purchased from eBioscience. Biotinylated HABP (hyaluronan binding protein, HABP) was from Merk (Darmstadt, Germany). Mouse anti-CD44 (clone: 515) was obtained from BD Biosciences Pharmingen (San Diego, CA). Native high-molecular-weight hyaluronan was obtained from Sigma-Aldrich (St. Louis, MO, USA). 1,2-Dilauroyl-sn-Glycero-3-Phosphoethanolamine (DLPE) and 1,2-Dilauroyl-sn-Glycero-3-Glycerol (DLPG) were purchased from Avanti Polar Lipids Inc. (Alabaster, AL, USA). PTX, semisynthetic from Taxus sp., minimum 97%, and 1-Ethyl-3-(3-dimethylamino-propyl) carbodiimide (EDAC) were purchased from Sigma. Courain6 and Qdot 800 were purchased from Invitrogen. All other chemicals were of reagent grade or higher.

### Immunohistochemistry

For immunostaining, frozen sections (8 μm) were prepared, fixed in acetone, air dried, and then stored at −80°C. After abolishment of endogenous peroxidase activity (0.3% H2O2, 20 minutes), tissues were rehydrated with PBS and blocked with 3% BSA in PBS for 30 minutes. Then, sections were treated with primary antibodies specific for CD44 for 60 minutes at room temperature. Detection was achieved with compatible-conjugated secondary antibody (Invitrogen, CA, USA) and Horse radish-peroxidase–conjugated (HRP-conjugated) ABC amplification system.

### Cell culture

Normal cell lines, mouse fibroblast cell line NIH3T3 and monkey kidney cell line CV-1, were bought from American Type Culture Collection (ATCC) and cultured in RPMI-1640 medium. The primary dermal fibroblasts in normal skin (NFs) derived from 3 adults were obtained and cultured as previously reported [20]. Human peripheral blood mononuclear cells (PBMC) were harvested using Ficoll density gradient centrifugation. Ficoll-Paque PLUS (GE Healthcare Life Sciences, Chalfont St Giles, UK) was overlaid with 5mL EDTA blood and centrifuged at room temperature for 20 minutes at 500g. Cells were then collected, washed twice with phosphate buffered saline (PBS). Four human breast tumor cell lines (BT549, MDA-MB-231, MDA-MB-468 and T47D) were obtained from ATCC and cultured in Dulbecco's Modified Eagle Medium (DMEM) supplemented with 10% fetal calf serum, 100 U/ml penicillin, and 100 mg/ml streptomycin. All cells were grown to 85% confluency for experiments.

### Preparation of HA-coated nanoparticles

Nanoparticles preparation was performed as our previously reported protocol with some modifications(21). HA was dissolved in acetate buffer (0.1M, pH 4.5) to a final concentration of 2 mg/ml and pre-activated by incubation with EDACat the concentration of 40 mg/ml for 2 h at 37°C. PTX and lipids (DLPE: DLPG mole ratio of 7:3) were dissolved in ethanol separately, then mixed together. Drug loading was at 1:4 drug-lipid (w/w). The solution was evaporated to dryness under reduced pressure in a Buchi Rotary Evaporator Vacuum System, hydrated by sterile water, then heated at 45°C for 2 h and sonicated for 10 min in a bath sonicator. The activated HA was added to the lipid-drug suspension with pH adjusted to 9.0 by adding NaOH and incubated over night at 37°C. Excess reactive agents and by-products were removed by extensive dialysis against ddH2O, pH 8.2.

### CD44 expression analysis in cell lines

Cultured cells were harvested and washed with washing buffer (phosphate-buffered saline (PBS) supplemented with 2% bovine serum albumin, pH 7.4). A single cell suspension (10^6^ cells/ml) was incubated with specific antibodies or isotope control antibodies on ice for 1 h. Cells were washed three times with washing buffer, and 1 μl of the anti-CD44 antibody was added for 1 h on ice. Cells were analyzed using a flow cytometer (Beckman-Coulter, Brea, USA), and at least 6,000 cells were analyzed per sample. All experiments were performed at least twice.

### CD44 activation analysis in cell lines

Fluorescein-labeled HA (fl-HA) binding was used to determine the CD44 activation state. Cells were incubated with 40 μg/ml fl-HA (Merck, Darmstadt, Germany) for 1 h at 4°C as described previously [22]. Then, the cells were treated with ethylenediaminetetraacetic acid (EDTA) and washed three times in PBS. After fixation with 1% paraformaldehyde, plasma membrane-bound fl-HA was analyzed using a flow cytometer (Beckman-Coulter, Brea, USA). Cancerous and normal cells were washed with PBS. Cells were incubated with 40 μg/ml fl-HA for 1 h at 4°C. After washing with PBS, plasma membrane-bound fl-HA was analyzed using a flow cytometer.

### Quantification of the expression of CD44 variants

Cells were harvested and total RNA was purified using TRIzol reagent. For reverse transcription, a random nonamer-oligo dT combination was used as the primer. To assess the variable CD44 regions, a PCR reaction was performed with a human-specific primer pair (forward primer: 5′-AGT CAC AGA CCT GCC CAA TGC CTT T, reverse primer: 5′-TTTGCT CCA CCT TCT TGA CTC CCA TG-3′ [25]. The PCR reaction mixture contained 5 μl 10 × PCR buffer with Mg^2+^ (Takara), 8 μl dNTP mix (2.5 mM each), 2.5 μl DNA polymerase (5 U/μl), 2 μl of each primer, 2 μl cDNA and 28.5 μl sterilized water for a final volume of 50 μl. The cycling conditions were 95°C for 5 min; 95°C for 30 sec, 52°C for 1 min, and 65°C for 2 min for 40 cycles; and an extension step at 65°C for 5 min. PCR products were separated on a 1.5% agarose gel and detected with a Gel Doc 2000 (Bio-Rad) after ethidium bromide staining.

Total cell lysates were collected, and equal quantities of protein were separated via 8% sodium dodecyl sulfate polyacrylamide gel electrophoresis (SDS-PAGE) and blotted onto a polyvinylidene difluoride (PVDF) membrane. PVDF membranes were blocked with Tris-buffered saline (TBS) containing 5% skimmed milk powder for 1 h, washed for 1 h in TBS, and incubated with CD44 (156–3C11, Cell Signal) or β-tubulin monoclonal antibody (mAb, Cell Signal) at 4°C for 3 h. Then, the membranes were washed with TBS/Tween 20 (TBS/T) buffer three times (5 min each time) and incubated with horseradish peroxidase (HRP)-conjugated polyclonal secondary antibody for 1 h. The membranes were developed using the enhanced plus chemiluminescence assay (Thermo, USA) according to the manufacturer's instructions. Images were analyzed using Image Pro-Plus 6.0.

### Tumor xenograft mouse model

All protocols involving mice were evaluated and approved by our Institutional Animal Care and Use Committee and performed under veterinary supervision. The breast cancer xenograft model was established in nude mice by injecting MDA-MB-231 cells as previously described [23]. Nude mice at 5 to 6 weeks of age received a subcutaneous injection of 1.0 × 10^6^ MDA-MB-231 cells. Tumor growth was monitored by palpation, and the date of detectable tumor onset was noted. Tumor size was measured with calipers, and tumor volume was calculated based on the assumption of an ellipsoid shape. Representative data were obtained from five mice per experimental group, and the entire experiment was repeated in three independent trials. Mice received intravenous injections of PBS (as a negative control), taxol (as a positive control), HA-Lipid-PTX, or HA-free Lipid-PTX 4 times every 5 days. Tumor growth and mouse body weight were monitored until day 30, at which time the mice were sacrificed, and the tumor tissues were excised for histological analysis.

### *In vivo* targeting characteristics and biodistribution of HA-coated nanoparticles

The biodistribution and tumor accumulation profiles in live animals were imaged using the IVIS Imaging system (Xenogen 200). A 670 nm-pulsed laser diode was used for the excitation, and the Qdot800 fluorescence images were obtained using a 790-nm emission filter. To estimate the time-dependent excretion profile, the total Qdot fluorescence intensity per selected region (1000 mm^2^) in the entire body was calculated as a function of time. The tumor targeting characteristics of HA-NPs were evaluated by measuring the Qdot fluorescence intensity at the tumor site (40 mm^2^). All data were calculated using the region of interest (ROI) function in the Analysis Workstation software (ART Advanced Research Technologies Inc., Montreal, Canada), and values are presented as the mean ± SE (standard error) for groups of three animals.

Major organs and tumors were dissected from MDA-MB-231 tumor-bearing mice 24 h after intravenous injection of HA-coated nanoparticles. Fluorescence images of dissected organs and tumors were obtained using an IVIS imaging system. The tissue distribution of nanoparticles was quantified by measuring Qdot800 fluorescence intensity at the ROI. All values are expressed as the mean ± SE for groups of three animals.

### Statistical analysis

CD44 expression and activation levels were analyzed by integral optical density (IOD) values with Image Pro Plus V6.0 for Windows (Media Cybernetics, Inc., Rockville, MD, USA). Briefly, after intensity rectification, IODs were obtained as the ratio of the sum optical density (OD) to the sum area. Most of the data were expressed as a median or a range, and the median was presented as the mean staining intensity (MSI). Mann-Whitney and Kruskal-Wallis tests were used to determine whether the association between activated CD44 and breast cancer progression was modified by other factors. The following covariates were included in the analyses: tumor grade (categorized as grade 1, 2, or > grade 3), histological type (categorized as invasive duct carcinoma vs. non-invasive duct carcinoma), tumor size (categorized as a grade 1, 2, or > grade 3), lymph node metastasis stage (categorized as no vs. yes), age at diagnosis (categorized as < 50 years vs. ≥ 50 years), estrogen receptor status (categorized as positive vs. negative), progesterone receptor (categorized as positive vs. negative), HER2 (human epidermal growth factor) (categorized as positive vs. negative), and Ki 67 (categorized as < 20% vs. ≥ 20%). Statistical significance in this study was set at *P* < 0.05, and all of the reported P-values are 2 tailed. All analyses were performed with SPSS v.16 for Windows (SPSS Inc., Chicago, IL, USA) or SigmaPlot v12 for Windows (Systat Software, Inc., San Jose, CA, USA).

## SUPPLEMENTARY MATERIAL FIGURE



## Abbreviations

HA: hyaluronan
PTX: Paclitaxel
Qdot: quantum dot
HA-Lipid-PTXs: HA-coated nanoparticles
NFs: The primary dermal fibroblasts in normal skin
