# Supplementary material for: Selective killing of breast cancer cells expressing activated CD44 using CD44 ligand-coated nanoparticles in vitro and in vivo
Source: Oncotarget. 2015 Mar 29;6(17):15283–96. doi: 10.18632/oncotarget.3681 (PMC4558151; doi:10.18632/oncotarget.3681)
Supplement: Supplementary file 1 [file oncotarget-06-15283-s001.pdf]

Selective killing of breast cancer cells expressing activated CD44 using CD44 ligand-coated nanoparticles *in vitro* and *in vivo*

Supplementary Material

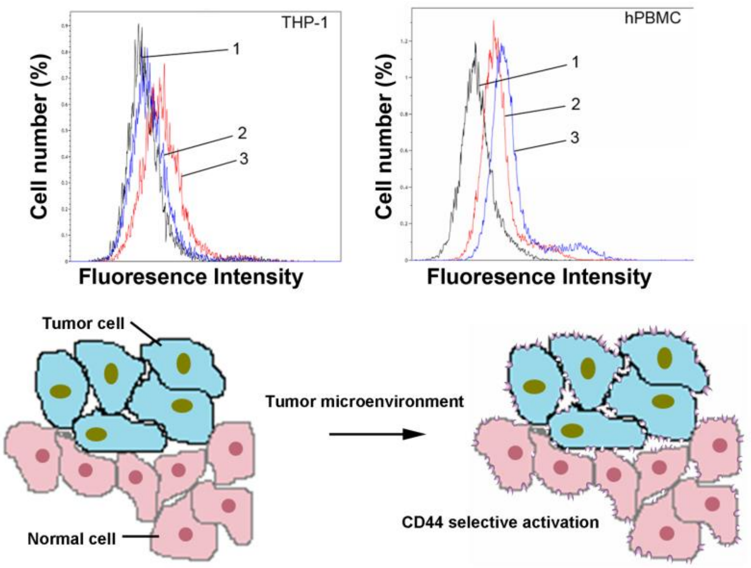

Fig.S1: CD44 is activated in the tumor microenvironment.

THP-1 cells and human peripheral blood mononuclear cells (PBMC) were co-cultured with or without the human breast cancer cell line BT549 at 37°C for overnight. Cells were collected, centrifuged, washed, and resuspended in phosphate-buffered saline (PBS) with 0.1% BSA. Cells were incubated with 40 µg/ml fl-HA (Merck) for 1 h at 4°C, and then the cells were washed three times in PBS. After fixation with 1% paraformaldehyde, plasma membrane-bound fl-HA was analyzed using a flow cytometer (Beckman-Coulter, Brea, CA). CD44 was activated in the tumor microenvironment. 1, Naïve cells cultured in complete medium. 2, Cells co-cultured with BT549. 3, Cells stimulated with phorbol 12-myristate 13-acetate (PMA).
